# Is vitamin D supplementation program in Iranian schools effective in reducing adolescent depressive symptoms? Cost effectiveness study

**DOI:** 10.1186/s12889-023-16244-z

**Published:** 2023-07-20

**Authors:** Seyed Vahid Jasemi, Zhale Zandieh, Narges Zandieh, Mohsen Rezaei Hemami, Ali Darvishi, Zahra Abdollahi, Ramin Heshmat

**Affiliations:** 1 Clinical Research Development Unit, Imam Reza Hospital, Kermanshah University of Medical Sciences, Kermanshah, Iran; 2 Aging Research Center, Department of Gerontology, Faculty of Rehabilitation, University of Welfare Sciences and Social Health, Tehran, Iran; 3 Health Economist at Perspectum Ltd, Oxford, UK; 4 Department of Management and Health Economics, School of Public Health, Tehran University of Medical Sciences (TUMS), Tehran, Iran; 5 Chronic Diseases Research Center, Endocrinology and Metabolism Population Sciences Institute, Tehran University of Medical Sciences, Tehran, 1941933111 Iran; 6 Office of Community Nutrition, Deputy of Health, Iran Ministry of Health and Medical Education, Tehran, Iran

**Keywords:** Vitamin D supplementation, Cost-effectiveness Analysis, Depression, Adolescents, Iran

## Abstract

**Purpose:**

We aimed to assess the cost-effectiveness of the vitamin D supplementation program in Iranian adolescents reducing adolescent depressive Symptoms.

**Methods:**

In the current cost-effectiveness analysis, the viewpoint of Iran’s Ministry of Health was selected. The target population was 1,519,762 Iranian high school students (733,657 girls and 786,105 boys). The total costs of the vitamin D supplementations program were based on the reports of the Nutrition Improvement Office of Iran’s Ministry of Health and were adjusted to 2018. The variable of Quality-Adjusted Life Years (QALYs) was considered a suitable variable for estimating the effectiveness of vitamin D supplementation. We chose one year as the time horizon. A decision tree model was constructed in TreeAge Pro. The results of the cost-effectiveness analysis were reported in term of the Incremental Cost-Effectiveness Ratio (ICER).

**Results:**

The results of our study showed that the estimated cost per QALY gained of the vitamin D supplementation program is equal to 1528.6676 $, which indicates that vitamin D supplementation in adolescents(11-18Y) is a cost-effective and a dominant strategy in preventing depression through the cost-saving and QALYs increment compared to the no intervention. Sensitivity analysis showed that the possible variations in vitamin D supplement costs could not alter the results, and vitamin D supplementation may be a predominant and cost-effective strategy to prevent adulthood depression with a 100% probability.

**Conclusion:**

The national program of vitamin D supplementation among Iranian adolescents was a cost-efficient strategy reducing adolescent depressive Symptoms through the cost-saving and QALYs increment compared to the no intervention.

## Introduction

Depression is a major mental disorder worldwide, which is the leading cause of the overall global burden of disease [1, 2]. About five to eight percent of the total disability-adjusted life years (DALYs) in the middle- and high-income countries are related to depression [3, 4]. The prevalence of depression is increasing worldwide, and 264 million people of all ages are affected by depression based on the WHO reports up to 2020 [5, 6]. The exact pathophysiology of depression is poorly understood, but there is now compelling evidence that vitamin D has a major role in brain and depression development [7, 8]. The different distribution of this ligand’s receptor and the final catabolic enzyme in its synthesis in the human brain raises several issues about the steroid’s function in the adult brain [9]. A number of potential roles for Vitamin D in the brain have recently been hypothesized, including the possibility of Vitamin D acting similarly to neuroactive hormones by influencing neuronal excitability. Moreover, multiple studies have revealed that vitamin D can influence the production of particular neurotrophins [9, 10]. Considering the role of vitamin D in brain processes, it seems that vitamin D deficiency causes depression [11]. Based on the findings of the previous studies, vitamin D insufficiency is prevalent among patients with fibromyalgia and is particularly prevalent in those who suffer from anxiety and depression [12, 13].

Moreover, daily intake of 2,460 IU vitamin D for 28 days on 113 adolescents and children with at least mild depression can improve the serum levels of vitamin D, and also the parents of patients receiving vitamin D have reported a significant reduction in their children’s depressive symptoms [14]. On the other hand, high-dose vitamin D supplementation (receiving 50,000 IU vitamin D weekly for nine weeks) in 9,000 adolescent girls or 200,000 IU at first and then 25,000 IU every two weeks for four months in 158 adolescent girls with vitamin D deficiency were associated with a significant reduction in mild, moderate, and severe depression scores, or anxiety, irritability, and sadness, respectively [15, 16].

Due to the high prevalence of vitamin D deficiency in Iranian children and adolescents for several reasons [17, 18], Iran’s Ministry of Health conducted a national vitamin D supplementation program in 2014 to correcting the vitamin D deficiency in Iranian adolescents.

Since performing a national supplementation program can impose a heavy burden on the economy and healthcare system, and also the results of the previous studies have shown that vitamin D deficiency is associated with depression sign [19–21], as well as the intake of vitamin D supplements in different doses in adolescents can reduce the symptoms of depression [14–16], so it would be valuable to examine the cost-effectiveness of such programs. Therefore, the current cost-effectiveness analysis was done in a period of one year in the age group of Iranian adolescents in school(11-18y) have been covered by the vitamin D supplementary national program toassess the cost-effectiveness of improve level of vitamin D versus without intervention in Iranian adolescents to reduce depression symptoms.

## Materials and methods

### National program’s characteristics

#### Target population and setting

In the current study, the target population was 1,519,762 students (733,657 girls and 786,105 boys) studying in the first and second grades of high school and from 47 different geographical regions of Iran who were covered by the National Vitamin D supplementation program in 2018. Their crude data were obtained from the Nutrition Improvement Office of the Ministry of Health. Of the 1,519,762 randomly selected individuals, 78.5% of the total population, 1,185,211 students, received vitamin D supplements.

#### Supplementation strategy

According to the protocols of this supplementary program, nine pearls of 50,000 IU vitamin D were considered for each student during the nine months (equivalent to the monthly consumption of one pearl). This supplementary program was performed for nine months in the fall, winter and spring seasons. In summer, due to the absence of students and also to prevent the risk of vitamin D toxicity, the vitamin D supplementation program was not implemented.

### Study perspective

Since the vitamin D supplementation program was a national program and performed by the Ministry of Health and Medical Education, we conducted our cost-effectiveness analysis from the MOHME perspective to determine the cost-effectiveness of a nationwide vitamin D supplementation among Iranian adolescents to prevent adulthood depression.

### Comparators and time horizon

Using a cost-effectiveness analytical approach, we compared two strategies of vitamin D supplementation and no intervention to capture whether the national vitamin D supplementation program for Iranian adolescents is a cost-effective treatment to reduce the risk of depression development in adulthood. We chose one year as the time horizon because the CASPIAN-III study’s vitamin D supplementation sessions lasted one year. In this way, vitamin D supplementation carried out 9 months of the year and 3 months of the year when sufficient sunlight was available no supplemental program was performed; Therefore, to ascertain the cost-effectiveness of the monthly intake of 50,000 IU vitamin D for nine months in Iranian adolescents, we set the time horizon of one year to eliminate errors and biases.

### Health outcomes and measurement

To determine the effectiveness and benefits of the Vitamin D supplement program in preventing depression, we measured Quality-Adjusted Life Year (QALYs) values for healthy individuals and patients with depression. In our study, we calculated the average of the utility value estimates in patients with depression [22, 23] or healthy ones [24, 25] based on the published studies. Moreover, serum levels of vitamin D less than 30 ng/ml and 30 or more than 30 ng/ml were considered as vitamin D deficiency or sufficiency, respectively [26]. The possible association of different serum levels of vitamin D with self-reported depression was obtained based on a recent study conducted on the CASPIAN-III study [19].

### Costs estimation

The estimate the total costs of the national vitamin D supplementation program, we need to consider both direct and indirect medical costs. The total costs of purchasing nine pearls for 50,000 IU of vitamin D per student is considered as the direct medical costs, while the indirect medical costs refer to the required costs to train project implementers, performing the program, and monitor how the program is done. In this way, the Nutrition Improvement Office of Iran’s Ministry of Health provided us with the required data, and we adjusted it to 2018.

According to the exchange rate of 9.6 reported by the Central Bank of the Islamic Republic of Iran in 2018, the total estimated project costs were converted to US dollars (USD) [27].

### Model and analyses

**Fig. 1:**
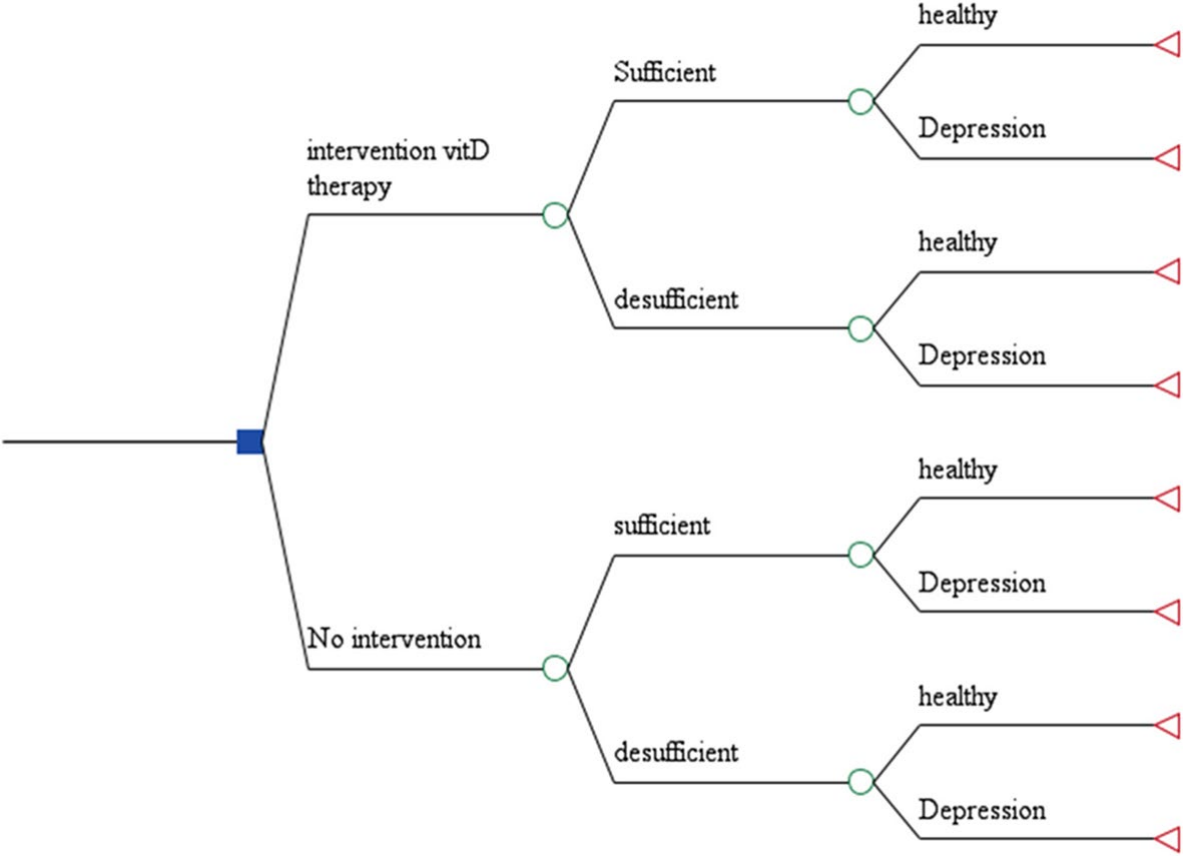
The decision tree model diagram (vitamin D intervention vs. no intervention)

The two strategies of vitamin D supplementation and no intervention were compared in terms of cost and effectiveness (Fig. 1), and the results were reported in the form of the Incremental Cost-Effectiveness Ratio (ICER). ICER was calculated by dividing the difference in total costs (incremental cost) by the difference in the chosen measure of health outcome or effect (incremental effect) to provide a ratio of ‘extra cost per extra unit of health effect’ – for the more expensive therapy vs the alternative [28].

$$=\frac{\mathrm{Costs}\, \mathrm{of\, intervention}-\mathrm{Costs\, of\, no\, intervention}}{\mathrm{QALYs\, of\, intervention}-\mathrm{QALYs\, of\, no\, intervention}}$$

The ICER reveals the cost of vitamin D supplementation (compared to no supplementation) per each QALYs gained and can be calculated as the differences between the total costs of vitamin D supplementation and no intervention (∆ costs) divided by the difference between the related values of QALYs for them (∆ QALYs). The QALYs was also considered as a suitable variable for estimating the effectiveness [29].

To discuss that the national program of vitamin D supplementation can be a cost-effective strategy in depression prevention than no intervention, the ICER value was compared with the maximum willingness to pay (WTP) per QALY as a cost-effectiveness threshold. Based on the related study about the estimated WTP for QALY in Iran [30], we considered the range of $1032–2666 per QALY as the threshold of cost-effectiveness.

We constructed the decision tree model in Microsoft Excel and programmed it through TreeAge Pro Inc. 2011 software (TreeAge Software, Williamstown, Massachusetts, USA).

### Sensitivity analysis

In order to assess the uncertainty of the evaluation results, we performed a sensitivity analysis. According to the variations in costs of vitamin D pearls, treating depression, and the estimated values of QALYs for healthy individuals and patients with depression reported by the previous articles, we performed a probabilistic sensitivity analysis (PSA) using Monte Carlo simulation to show whether the mentioned variations can affect the cost-effectiveness of the vitamin D supplementation program in preventing depression or not [28, 31].

## Results

### Effectiveness

As mentioned, to determine the effectiveness of vitamin D supplementation, the amount of QALYs was considered the target variable. Based on the previous studies, the suggested QALY values for moderate and severe depressed people were about 0.57(95% CI, 0.54–0.61) and 0.52(95%CI, 0.49–0.56), respectively [23]. Therefore, we considered the average of these utility scores as the QALY value for depression, equal to 0.545(95%Cl, 0.51–0.58). On the other hand, the QALY value for the healthy individual was considered equivalent to 0.76 [24, 25].

The previous studies showed a significant prevalence of vitamin D deficiency among the Iranian children. In 2014, Rafraf conducted a study on 216 adolescent girls and showed that 96% of the study population had vitamin D deficiency and the mean serum level of vitamin D was 7.26 (sd 2.81) ng/ml [32]. In a nationwide cross-sectional study on 1,095 Iranian adolescents aged 10–18 years, Kelishadi and her colleagues found that 40% of participants including 40.70% of boys and 39.30% of girls had vitamin D deficiency [33]. Moreover, according to a study conducted by Heshmat et al. in 2008 on 5,232 people from five urban metropolitans’ cities in Iran, the prevalence of vitamin D deficiency among the study population was 76%, and only 24% had adequate levels of vitamin D [18]. Based on the recent meta-analysis, the pooled prevalence of vitamin D deficiency in Iranian children was about 60.10% [17].

According to the CASPIAN-III study [19], the prevalence of self-reported depression was about 16% among adolescents with adequate vitamin D levels and 31.4% among adolescents with inadequate vitamin D levels (as shown in Table 1). Based on this, it can be concluded that insufficient serum levels of vitamin D can double the prevalence of depression.

**Table 1:** Main input parameters of CEA model

**Statistic variable**	Base case	SD/(CI)	Distribution	Source
***Costs($)***				
Total costs of vitamin D therapy	0.604	± 0.120	Gamma	^a^
The 2018-adjusted costs^b^ of depression management for each patients^c^	335.4	± 35	Gamma	[34]
Direct medical costs of the vitamin D supplementation national program for each student	0.354	± 0.095	Gamma	^a^
Indirect medical costs of the vitamin D supplementation national program for each student	0.250	± 0.025	Gamma	^a^
***Utilities***				
Individuals without depression	0.760	± 0.070	Beta	[25, 35]
Individuals with depression	0.545	± 0.035	Beta	[22, 23]
***Probability***				
The possibility of vitamin D deficiency before starting vitamin D supplementation	76%	*-*	*-*	[18]
The possibility of vitamin D sufficiency before starting vitamin D supplementation	24%	-	-	[18]
The possibility of vitamin D deficiency after starting vitamin D supplementation	17.2%			[36]
The possibility of vitamin D sufficiency after starting vitamin D supplementation	87.2%			[36]
The prevalence of depression incidence in people with vitamin D deficiency	31.4%			[37]
The prevalence of depression incidence in people with vitamin D sufficiency	16%			[37]

^a^the Nutrition Improvement Office of the Ministry of Health

^b^According to Iran’s Central Bank reports, the inflation rates in 2018 was 9.6

^c^The expenditure data in 2018 was calculated at a dollar exchange rate declared by Iran’s Central Bank

Because of high prevalence of vitamin D deficiency in Iranian adolescents, it is necessary to implement the supplementary strategies. While the monthly intake of 60,000 IU vitamin D orally for one year in children can reduce the prevalence of vitamin D deficiency by up to 17.2% [36]. Thus, it can be proposed that vitamin D supplementation in adolescents, by improving serum vitamin D status, might be an effective strategy in reducing the prevalence of adulthood depression.

### Estimated costs

The cost of treating depression for each patient in 1993 was estimated at 988,195 Iranian Rials [38], which is equivalent to 14,087,678 Iranian Rials in 2018 according to the Iran’s inflation rate. By considering Iran’s exchange rate reported by the Central Bank of the Islamic Republic of Iran in 2018, the 2018-adjusted costs of treating depression would be equivalent to 335.4 USD for each patient.

The total costs of performing the National Program of vitamin D supplementation per student in 2018 have been estimated to 20,957 to 30,865 Iranian Rials (0.499–0.72 USD). As mentioned in the methodology section, project costs consist of the sum of direct and indirect medical costs. According to the MOHME reports in 2018, direct medical costs (to purchase nine pearls of vitamin D) per student ranged from 9,908 to 19,816 Iranian Rials (equivalent to 0.236–0.472 USD). Also, the indirect medical costs associated with running and supervising a project per student were estimated at 11,049 Iranian Rials (0.25 USD) (as shown in Table 1).

### Cost-effectiveness analysis

The results of cost-effectiveness analysis showed that the estimated ICER of the vitamin D supplementation program is equal to 1528.6676 $. The estimated cost per QALY gained value indicates that the vitamin D supplementation n in adolescents is a cost-effective and a dominant strategy in preventing depression through the cost-saving and QALYs increment compared to the no intervention (Table 2).

**Table 2:** Results of base case cost-effectiveness analysis

**Strategy**	**Cost($)**	**Incr Cost($)**^a^	**Eff**^b^	**Incr Eff**^c^	**ICER($)**
No Intervention	92.91	29.76	0.70	-	1528.66
Vitamin D Supplementation	**63.15**	**0**	**0.71**	**0**	

^a^Incremental costs

^b^Effectiveness

^c^Incremental Effectiveness

### Sensitivity analysis

**Fig. 2:**
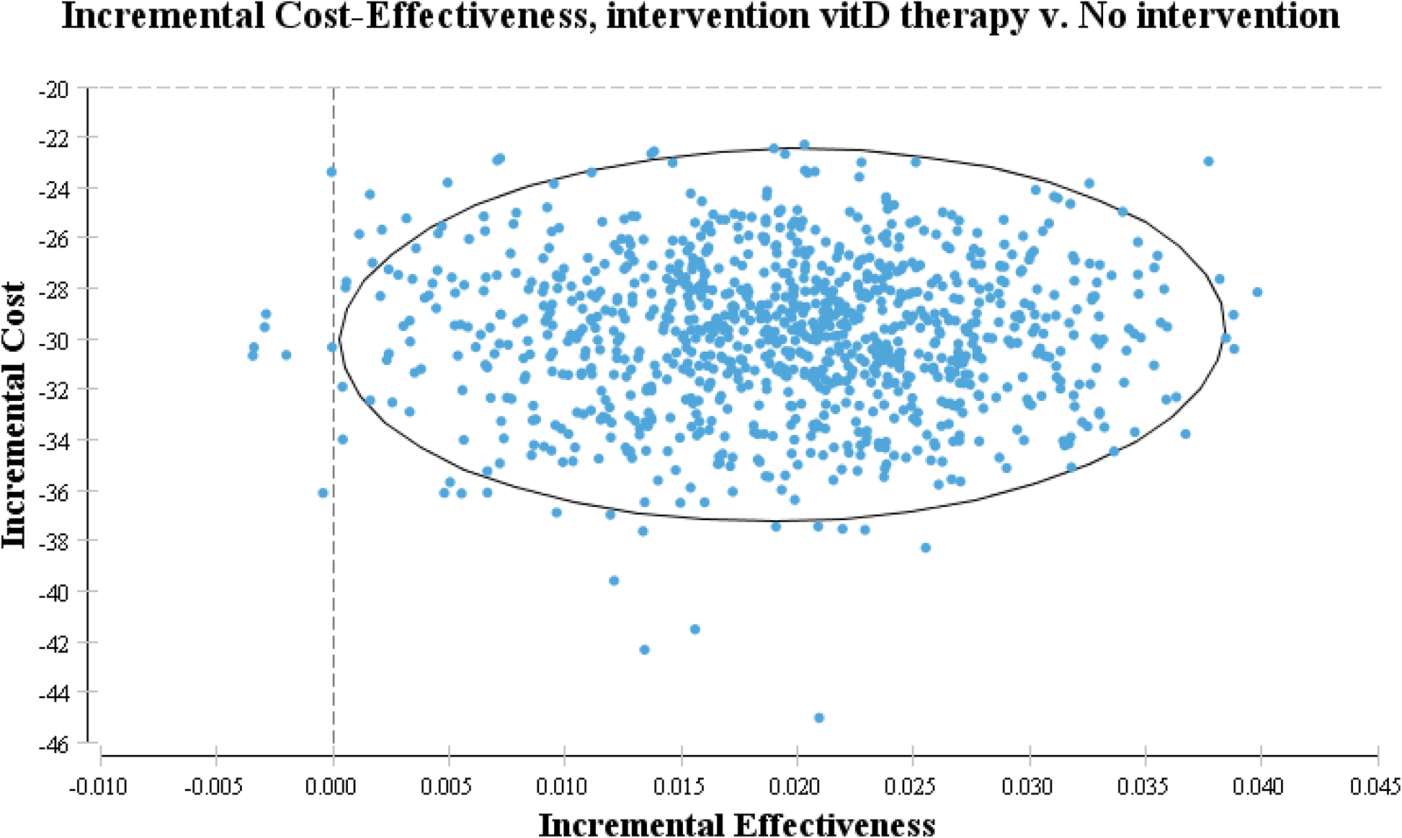
The incremental cost-effectiveness scatter plot (all samples are in the acceptable range)

**Fig. 3:**
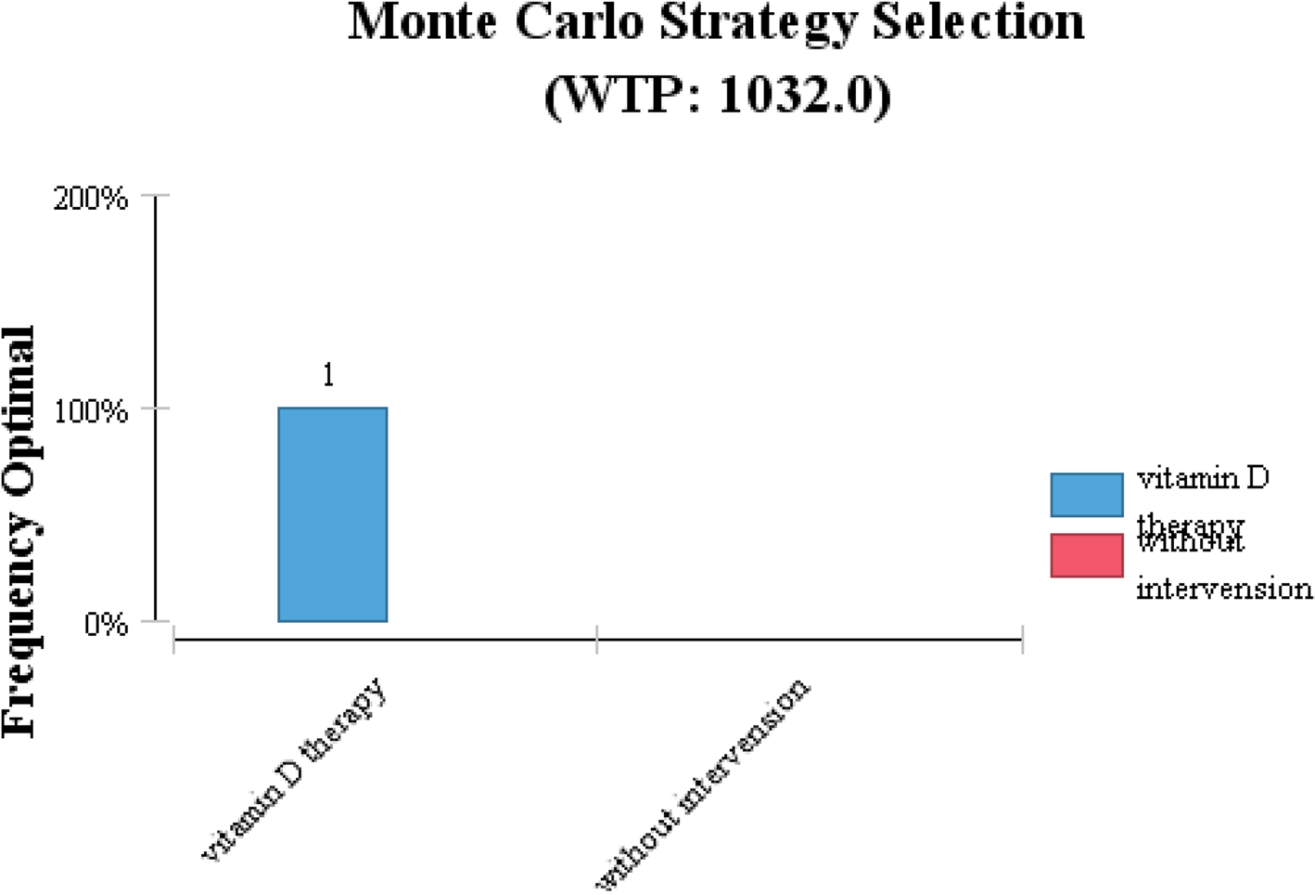
The Monte Carlo Strategy Selection

The results of cost-effectiveness analysis showed that the estimated ICER of the vitamin D supplementation program is equal to 1528.6676 $. After performing the sensitivity analysis, the ICER value of vitamin D supplementation was not sensitive to the variation (Table 1). As shown in Fig. 2, all samples are in the acceptable range, and based on the results of the Monte Carlo simulation, vitamin D supplementation can be a cost-effective strategy to prevent adulthood depression with a 100% probability compared to the no intervention (Fig. 3).

## Discussion

The current study revealed that the national program of vitamin D supplementation among Iranian adolescents was a cost-effective (11-18y) strategy to prevent depression in adulthood through the cost-saving and QALYs increment compared to the no intervention. Also, by considering the possible variations in vitamin D supplement prices, the findings of sensitivity analysis showed that the results are not sensitive to parameter variations, and vitamin D supplementation may be a cost-effective strategy to decrease depression symptoms in adolescent with a 100% probability.

Based on our findings, no vitamin D supplementation can increase the prevalence of vitamin D deficiency among adolescents by up to 4.5 times, and vitamin D deficiency can increase the risk of mild to moderate depression by up to 1.9 times. It seems that vitamin D supplementation can reduce the risk of depression by improving the serum level of vitamin D. There is strong evidence suggesting an inverse association between serum levels of vitamin D and emotional problems, especially depression [20], and the prevalence of depression was significantly lower among people who were not vitamin D deficient [19, 21]. In this regard, the previous studies suggested that vitamin D supplementation is recommended as one of the effective factors in preventing depression. The intake of vitamin D supplements are related to a significant reduction in the children’s depressive symptoms [14], a significant reduction in mild, moderate, and severe depression scores [15], and a significant reduction in anxiety, irritability, and sadness in 158 adolescent girls with vitamin D deficiency [16].

According to the CASPIAN-III study [19], Iranian adolescents deficient in vitamin D were more likely to suffer from depression than the vitamin D sufficient people. So, it seems that the national program of vitamin D supplementation among Iranian adolescents may be an effective strategy in depression prevention through the serum levels of vitamin D correction [16]. By performing the decision tree model, it was found that the vitamin D strategy was related to lower costs and higher QALY than no intervention in depression treatment in adulthood. By considering the role of vitamin D supplementation in the risk reduction of mild to moderate depression and the high costs of treating depression, this can be suggested that vitamin D therapy may be a cost-saving strategy to prevent adulthood depression in comparison to no intervention. This cost-effectiveness was also no sensitive to variation in costs of vitamin D pearls and QALY values.

To the best of our knowledge, the current study is the first one that evaluated the cost-effectiveness of vitamin D supplementation in adolescents in depression prevention. We believed that the present study results could help healthcare policymakers better allocate financial resources and plan national depression prevention programs. In this study, we did not have access to data from adolescents who dropped out of school, and only student's data were evaluated. Fortunately, dropouts had a very small population, so that number could be negligible. More researches and economic evaluations in the general population are needed in the future to confirm our findings.

## Conclusion

This study suggested that the vitamin D supplementation national program among adolescents could be clinically a cost-effective strategy for decrease symptoms of depression among adolescent. Our results will potentially help the healthcare policymakers reduce the healthcare system costs by implementing the vitamin D supplementation national program among Iranian adolescents and better financial resources allocation.

## Data Availability

The datasets generated and analyzed during the current study are not publicly available but data can be retrieved from the authors, Ramin Heshmat with E-mail: r_heshmat@yahoo.com or Zahra Abdollahi With Email: Abdollahi_z@yahoo.com on reasonable request.
